# The human RAD52 complex undergoes phase separation and facilitates bundling and end-to-end tethering of RAD51 presynaptic filaments

**DOI:** 10.1093/nar/gkag043

**Published:** 2026-01-28

**Authors:** Ibraheem Alshareedah, Sushil Pangeni, Paul A Dewan, Masayoshi Honda, Ting-Wei Liao, Maria Spies, Taekjip Ha

**Affiliations:** Howard Hughes Medical Institute and Program in Cellular and Molecular Medicine, Boston Children’s Hospital, Boston, MA 02115, United States; Department of Pediatrics, Harvard Medical School, Boston, MA 02115, United States; Howard Hughes Medical Institute and Program in Cellular and Molecular Medicine, Boston Children’s Hospital, Boston, MA 02115, United States; Department of Biophysics, Johns Hopkins University, Baltimore, MD 21205, United States; Howard Hughes Medical Institute and Program in Cellular and Molecular Medicine, Boston Children’s Hospital, Boston, MA 02115, United States; Harvard Biophysics Graduate Program, Harvard University, Cambridge, MA 02138,United States; Department of Biochemistry and Molecular Biology, University of Iowa Carver College of Medicine, 51 Newton Road, Iowa City, IA 52242, United States; Howard Hughes Medical Institute and Program in Cellular and Molecular Medicine, Boston Children’s Hospital, Boston, MA 02115, United States; Department of Biophysics, Johns Hopkins University, Baltimore, MD 21205, United States; Department of Biochemistry and Molecular Biology, University of Iowa Carver College of Medicine, 51 Newton Road, Iowa City, IA 52242, United States; Howard Hughes Medical Institute and Program in Cellular and Molecular Medicine, Boston Children’s Hospital, Boston, MA 02115, United States; Department of Pediatrics, Harvard Medical School, Boston, MA 02115, United States; Department of Biophysics, Johns Hopkins University, Baltimore, MD 21205, United States

## Abstract

Human RAD52 is a prime target for synthetic lethality approaches to treat cancers with deficiency in homologous recombination. Among multiple cellular roles, RAD52’s functions in homologous recombination repair and stalled replication fork protection appear to substitute for those of the tumor suppressor protein BRCA2. However, the mechanistic details of how RAD52 substitutes for BRCA2 functions are only beginning to emerge. RAD52 forms an oligomeric ring enveloped by ∼200-residue-long disordered regions, forming a highly multivalent and branched protein complex that promotes supramolecular assembly. Here, we demonstrate that RAD52 undergoes homotypic phase separation, forming condensates that recruit key homologous recombination factors, including single-stranded DNA (ssDNA), replication protein A (RPA), and the RAD51 recombinase. Furthermore, we show that RAD52 phase separation is regulated by its interaction partners such as ssDNA and RPA. Through fluorescence microscopy, we observe that RAD52 promotes the formation of RAD51-ssDNA fibrillar structures. To resolve the fine architecture of these fibrils, we employed single-molecule super-resolution imaging via DNA-PAINT and atomic force microscopy, revealing that RAD51 fibrils comprise bundles of individual RAD51 nucleoprotein filaments. Additionally, we show that RAD52 induces end-to-end tethering of RAD51 nucleoprotein filaments. Collectively, these findings highlight distinctive macromolecular organizational features of RAD52 that may underpin its diverse cellular functions.

## Graphical Abstract

**Graphical Abstract:**
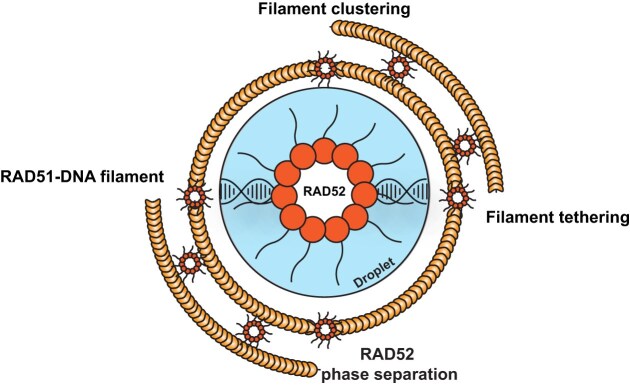

## Introduction

Genomic instability underlies many devastating diseases, including various types of cancers and other conditions associated with DNA damage [1–3]. It typically arises from errors in genome repair following exposure to genotoxic stress [1]. Among the forms of DNA damage, double-strand breaks (DSBs) are the most lethal and can occur during DNA metabolism or result from reactive oxygen species and irradiation [4]. DSBs are primarily repaired via non-homologous end joining (NHEJ) and homologous recombination (HR) [5]. HR preserves genetic information by restoring the original sequence using a homologous sequence elsewhere in the nucleus [6]. This pathway is most active during the G2/S phase, when sister chromatids are available to serve as repair templates [7].

For HR-mediated DSB repair, the DNA ends are initially processed by the MRN complex, a tri-protein complex involved in DNA resection [8]. Long-range resection is subsequently mediated by Exo1 or DNA2, generating ∼5 kb single-stranded (ss)DNA overhangs at the DSB ends [9, 10]. These single-stranded DNA (ssDNA) stretches are rapidly coated by replication protein A (RPA) to protect them from nucleolytic degradation [11, 12]. RPA is then replaced by RAD51, which forms a RAD51-ssDNA nucleoprotein filament (NPF) known as the presynaptic complex [13]. This filament performs a homology search in the genome, aided by cohesin-mediated loop extrusion anchored at the break [14, 15], to locate a suitable donor sequence for template-guided repair [13, 16]. Upon recognition, RAD51 mediates strand invasion into the homologous dsDNA donor, forming a D-loop that recruits DNA polymerases for synthesis based on the donor template [13]. Resolution of the D-loop and ligation of the repaired DNA complete the repair process [13]. In addition to its role in HR, the RAD51 NPF contributes to DNA replication by protecting stalled or damaged replication forks from degradation [17].

Many steps and mechanistic details of HR remain under extensive investigation. A central element in HR is the assembly of the RAD51 NPF [16], which requires the replacement of RPA on ssDNA with RAD51, a process facilitated by mediator proteins [18]. In humans, BRCA2 serves as the primary mediator, while Rad52 plays this role in yeast [19–21]. A recent study explored the mechanism of yeast Rad52 stabilizing Rad51 filament via binding-induced folding that is similar to BRCA2 in humans, which is considered its functional homolog [22]. Other studies probed the distinct modes of interactions between yeast Rad52 and yeast Rad51 [23, 24]. Interestingly, humans also possess RAD52, which shares several biochemical characteristics with its yeast counterpart; however, its mediator activity remains a subject of debate. Some *in vitro* studies suggest that human RAD52 lacks clear mediator activity during RAD51 NPF formation [19, 25], which is consistent with reports that RAD52 cannot fully compensate for BRCA2 deficiency in human cells [26]. Conversely, other studies demonstrate that RAD52 enhances D-loop formation [27, 28] and facilitates homology search [29], implying its role in HR may be context-dependent.

Historically, RAD52 was considered dispensable for human cell viability [25, 30, 31]. However, emerging evidence reveals a synthetic lethality relationship between RAD52 and BRCA2, indicating that RAD52 becomes essential when BRCA2 function is compromised [25, 32–35]. Notably, BRCA2 mutations are linked to various cancers and genetic disorders, such as Fanconi anemia [3, 36–39]. Therefore, inhibiting RAD52 kills cancer cells lacking BRCA2, while sparing normal cells, positioning RAD52 as a promising therapeutic target in BRCA-related malignancies [31, 40]. Understanding the molecular functions and behavior of RAD52 could thus pave the way for the development of novel cancer treatment strategies.

While the role of human RAD52 in HR remains unclear, studies have highlighted several additional functions of the protein. RAD52 possesses a ssDNA annealing activity, which the cell utilizes to repair DSBs committed to homology-directed repair but that cannot be resolved by HR [41, 42]. During S-phase, RAD52 plays two distinct roles at stalled DNA replication forks: initially, it protects the fork from reversal by motor proteins [43, 44], and subsequently, it cooperates with the MUS81 nuclease to cleave the stalled forks [45]. RAD52 is also required for the initiation of mitotic DNA synthesis and participates in telomeric DNA repair processes [46–48]. In telomerase-negative cancer cells, RAD52 is involved in the alternative lengthening of telomeres pathway [48, 49]. Further research has shown that RAD52 has high affinity toward G-quadruplex structures and R-loops and is able to mediate reverse strand exchange reaction involving both DNA and RNA [50–52]. In oncological contexts, RAD52 forms foci upon DNA damage. These foci are considered DNA repair centers, as they colocalize with other DSB repair proteins such as RAD51 [34, 35, 53–55]. RAD52 foci formation is also cell-cycle regulated, with increased foci formation observed during the G2/S phase, the same phase in which HR occurs [56]. Thus, in the absence of functional BRCA2, RAD52 may contribute to HR through self-assembly mechanisms that facilitate foci formation.

Despite renewed interest in the RAD52 biology and biochemistry, the mesoscale self-assembly properties of the human RAD52 complex remain poorly understood. Crystal structure and cryo-EM studies have shown that the human RAD52 assembles into either an undecameric ring or a decameric washer-like structure composed of 11 or 10 identical subunits [44, 57–61]. The N-terminal half of the protein harbors the oligomerization domain and two DNA-binding sites and is capable of being wrapped by ssDNA [62]. In contrast, the C-terminal domain is intrinsically disordered and contains binding sites for RPA and RAD51 [62–64]. This architecture results in a branched structure, with the ring encircled by 11 disordered regions, each ~200 amino acids in length, making RAD52 a compelling example of a branched multivalent protein complex. We propose that such multivalency may have significant implications for protein macromolecular assembly, enabling the RAD52 complex to bridge multiple molecules through its disordered C-terminal arms. Branched multivalency has been shown to confer unique condensation properties in other systems such as DNA nanostars [65]. Accordingly, the self-assembly and heterotypic interactions of RAD52 are particularly relevant from a polymer physics perspective.

In this work, we characterize the supramolecular self-assembly properties of RAD52 and its interactions with the RAD51 NPF and RPA. We first show that RAD52 undergoes phase separation at micromolar concentrations in aqueous environments and at nanomolar concentrations under molecular crowding conditions. These RAD52 condensates can recruit multiple key players in DSB repair, such as RPA, ssDNA, and RAD51. Due to its protein- and DNA-binding sites, RAD52 phase separation is highly sensitive to the presence of its interaction partners. We show that ssDNA facilitates RAD52 phase separation at low mixing ratios but inhibits it at high mixing ratios, consistent with a reentrant phase transition phenomenon [66]. In contrast, RPA enhances RAD52 phase separation in a monotonic fashion within our observation window. Furthermore, RAD51–ssDNA complexes form fibrillar structures spanning tens of microns in length upon the addition of RAD52. This occurs even in the presence of RPA, which is known to hinder RAD51–ssDNA interactions [67]. We hypothesized that these fibrillar structures correspond to bundles of RAD51 NPFs. Indeed, single-molecule imaging reveals that RAD52 induces clustering of individual RAD51 NPFs, facilitating fibril formation. Strikingly, RAD52 can tether RAD51 NPFs, resulting in exceptionally long filamentous assemblies. These observations are corroborated by atomic force microscopy data. Collectively, our results show that the human RAD52 complex undergoes phase separation and mediates both clustering and end-to-end tethering of RAD51 NPFs.

## Materials and methods

### Protein purification and fluorescence labeling

The human proteins 6×His-tagged-RAD52 [62], RAD52ΔCTD [62] (AA:1-212), RAD51 [68], and RPA [69] were expressed and purified to homogeneity using established protocols. The purified proteins were labeled via conjugating the N-terminal amine to an NHS ester conjugated fluorescent dyes (Lumiprobe) in a phosphate buffer (pH 7.0). The reaction was carried out at a 10:1 dye-to-protein ratio for 2 h at room temperature or overnight at 4°C. Ziba spin desalting columns (MWCO 7000) were used to separate the protein from the free dyes.

### Phase separation analysis

RAD52 was prepared in mixtures containing 25 mM Tris–HCl (pH 7.5) and 30 mM NaCl in the presence of various concentrations of polymer crowder PEG8000 (M.W. 8 kDa, Fisher Scientific). The samples were mixed and immediately placed on a Lab-Tek 8-well chamber cover glass for subsequent imaging. For RAD52–ssDNA mixtures, RAD52 was mixed with the appropriate amounts of ssDNA dT40-Cy3 (IDT) at a protein concentration of 2.5 µM in a buffer containing 25 mM HCl and 15 mM NaCl. For RPA–RAD52 mixtures, we prepared solutions containing 1 uM of RAD52, 1% (wt/vol) PEG8K, and variable RPA concentration in a buffer containing 25 mM Tris–HCl (pH 7.5) and 60 mM KCl. The salt identity and concentration were chosen partly based on the storage solution of the purified proteins, since buffer exchange may lead to complex dissociation and reduced protein stability. All samples were placed on a Nikon Eclipse Ti microscope equipped with a Nikon 100× oil immersion objective (Plan Apo 100×/1.4) and Andor iXon Ultra EMCCD camera. Each sample was imaged five times at different fields of view. For the multicomponent condensates preparation in Supplementary Fig. S4, we mixed RPA (1.2 µM + 300 nM RPA-MB543) and T30-ssDNA (with 200 nM Cy5-labeled 18mer DNA probe [GCCTCGCTGCCGTCGCCA]) in a buffer containing 25 mM Tris–HCl (pH 7.5), 30 mM NaCl, 40 mM KCl, and 1% PEG. We then added RAD52 to a final concentration of 5 µM.

### Recruitment experiments

Samples containing 5 µM concentration of RAD52 in 25 mM Tris–HCl (pH 7.5) and 30 mM NaCl in addition to nanomolar concentrations of a fluorescently labeled client [dT40-cy3, RPA-MB543, RAD51-AF 488, and telomeric repeat-containing RNA (TERRA) RNA-cy3] were added to the buffer prior to the addition of RAD52. The samples were placed into an eight-well Lab-Tek imaging chamber and loaded on a confocal microscope (Lumicks, Ctrap). Five images were taken for each sample for subsequent analysis of partition coefficients.

To calculate partition coefficients, we used ilastik pixel classification software [70], which relies on a neural network that can be trained manually by assigning pixel classifications. We manually selected pixels that exist within condensates and pixels within the background and trained a model that uses features like intensity, intensity gradient, Laplacian, and other features to determine droplet pixels and background pixels. The analysis was then done on the entire image sets (five images per sample) and outputs binary masks for the condensates. A custom-made Python script is then used to identify condensates and compute their mean intensities and the background intensity. The partition coefficient for every condensate is then calculated and averaged for the entire sample. Error bars are estimated by calculating the standard deviation of partition coefficients.

### RAD51 fibril sample preparation

Proteins and ssDNA were mixed in a 25 mM Tris–HCl (pH 7.5) and 100 mM NaCl buffer. The concentrations used for these experiments were 5 µM RAD51, 5 µM RAD52, 2.5 µM RPA, and 2.5 µM ssDNA. Different mixture combinations are shown in Fig. 4 of the main text. We found that the order of addition is critical for these experiments. To induce fibril formation, ssDNA is added first to the buffer. Then, RAD51 is added and mixed with ssDNA before other protein components are added (such as RAD52 or RPA). All samples contained nanomolar concentrations of fluorescently-tagged proteins. The samples were loaded onto an objective-type total internal reflection fluorescence (TIRF) microscope and imaged in TIRF/HiLo mode. The TIRF microscope is built on a Nikon Eclipse Ti frame equipped with an Apo TIRF 60x/1.49 oil immersion objective and an Andor iXon Ultra EMCCD camera. For excitation, three laser lines are used (Coherent OBIS) with wavelengths 488, 561, and 640 nm.

### DNA PAINT super-resolution imaging

RAD51 NPFs were prepared by mixing RAD51 and $\phi $X174 virion ssDNA at 2 µM RAD51 and 6 µM DNA (nucleotide concentration) in the reaction buffer, which contained 25 mM Hepes (pH 7.33), 30 mM KCl, 2 mM CaCl_2_, 2 mM ATP (adenosine triphosphate), and 2 mM TCEP (Tris-(2-Carboxyethyl)phosphine). The reaction was incubated for 15 min at 37°C. 0.1 µM RPA was added, and the reaction was further incubated for 45 min. The sample is then deposited in an eight-well Lab-Tek chamber coated with poly(lysine) (M.W. ∼1000–5000, P0879, Sigma–Aldrich) for 1 min and then rinsed off with phosphate buffered saline (PBS). The sample is then blotted and left to dry in a fume hood for 20 min. Dehydration allows for better attachment of the complexes to the surface. We also checked the same samples without the dehydration step and found no difference in the confirmation of filaments. Next, the sample is incubated with primary antibody against RAD51 (rabbit, PC130, Sigma–Aldrich) at 4°C overnight. For two-color experiments, primary antibodies for RPA (Mouse, MA1-26418, Thermo Fisher Scientific) or RAD52 (Mouse, MA5-31888, Thermo Fisher Scientific) were also added. Next, the sample was washed three times with PBS, followed by incubation of the oligo-conjugated secondary antibodies (Massive photonics, Fast sdAB 2-Plex kit) for 2 h at room temperature. The sample is then washed three times with PBS. Imaging solution is added with 0.5–3 nM of imager strand concentration. For RAD51, the imagers were conjugated with ATTO-655 dye (Massive Photonics). For the RAD52 and RPA, the imagers were conjugated with Cy3B (Massive photonics). The sample was then loaded on a home-built objective-type TIRF microscope with an Olympus IX71 frame, a 100×/1.4 PlanSApo Olympus oil immersion objective, and an Andor iXon EMCCD camera. The fluorescence illumination was achieved using a 500 mW 642 nm laser (MBP) and a 100 mW 568 nm laser (Coherent Sapphire) that were used to excite ATTO655 and Cy3B, respectively. The microscope had a steering lens that controlled the angle of illumination, which was set in TIRF mode. Movies were collected at an exposure time of 10 ms and for 10 000–20 000 frames. Image reconstruction, including fitting and rendering, was performed using the Picasso software from the Jungmann lab [71].

For strand invasion experiments, the homologous dsDNA ($\phi $X174 dsDNA RF1) was added and incubated for 30 minutes after the 1-hour-long assembly reaction had taken place. A similar procedure was performed for the RAD52-induced clustering with the following changes. RAD52 was added at 0.5 µM concentration and RPA was added at 1 µM at the start of the reaction. Then the reaction was incubated for 1 hr at 37°C before imaging.

### Atomic force microscopy imaging

#### Specification of high-speed atomic force microscope

A commercial sample-scanning high-speed atomic force microscope (HS-AFM) (SS-NEX Ando model) from RIBM (Research Institute of Biomolecule Metrology Co., Ltd.) was used for experiments involving RAD51 filaments and RAD51–RAD52 complexes. Tapping mode was employed to minimize interference with the deposited sample, and all deposited samples were captured in solution. Ultra-short cantilevers (USC-F1.2-k0.15-10), specifically designed for HS-AFM with a resonance frequency of 1200 MHz, a spring constant of 0.15 N/m, and a length of 7 µm, were purchased from NanoAndMore and utilized in these experiments. A wide scanner was employed with scan speeds ranging from 0.05 to 1 frame per second, with the resolution set to 200 × 200 pixels.

#### AFM sample preparation

RAD51 filaments in the presence and absence of RAD52 were prepared and incubated in the same way as described earlier for DNA PAINT imaging. A freshly cleaved mica surface was coated with poly(lysine) (M.W. ∼1000–5000, P0879, Sigma–Aldrich) at a concentration of 0.05 mg/ml. Poly-L-lysine was incubated for 3 min and washed off twice using distilled water. The RAD51 filament samples are diluted by a factor of 10 in the same buffer with ATP and then deposited on a freshly cleaved poly(lysine)-coated mica surface. After 10-min incubation, the sample is washed with distilled water and imaged with a high-speed AFM microscope.

#### Image processing and analysis

HS-AFM images were viewed and analyzed using the software built by Prof. Toshio Ando’s laboratory-built software, Kodec 4.4.7.39, with available source code [72]. Tilt and other image correction details are available in the literature [73]. Multiple frames were taken per structure and then projected with the median intensity value using Fiji-ImageJ software. The contrast was adjusted to enhance the structural features of the images.

### Quantification of phase separation from brightfield images

To quantify the degree of phase separation from brightfield images, we used image analysis. Each sample was imaged five times at different fields of view within the solution (away from the surface). Each brightfield image was processed with a Farid filter, which is an image derivative filter that calculates intensity fluctuations in the XY plane. The resulting filtered image contains pixel values proportional to the gradient of intensity in the pixel. We then calculate the standard deviation of the pixel values in the filtered image. The filtered image is then passed through a logical filter with the condition (pixel value >10 standard deviations), which will select the pixels containing high values of the image derivatives, which corresponds to sharp edges (i.e. edges of condensates in focus). The out-of-focus droplets are filtered out since the gradient of intensity will be lower than those that are in focus. This filtering of edges ensures that the background variations in intensity that could come from deposits on the surface and/or optical aberrations are not included in the calculations. Lastly, the number of selected pixels corresponding to the edges of droplets in focus is counted and divided by the area of the field of view, giving an estimate of the area fraction of droplet edges. This analysis is done for all five images, and the resulting edge fractional area is averaged to give a quantitative indicator of the degree of phase separation. The errors are calculated as the standard deviation of the edge fractional areas of the five images. All the numbers are then normalized with the value obtained for a non-phase separating sample to remove the contribution of speckles within the microscope optics.

## Results

### RAD52 undergoes homotypic phase separation

**Figure 1.**
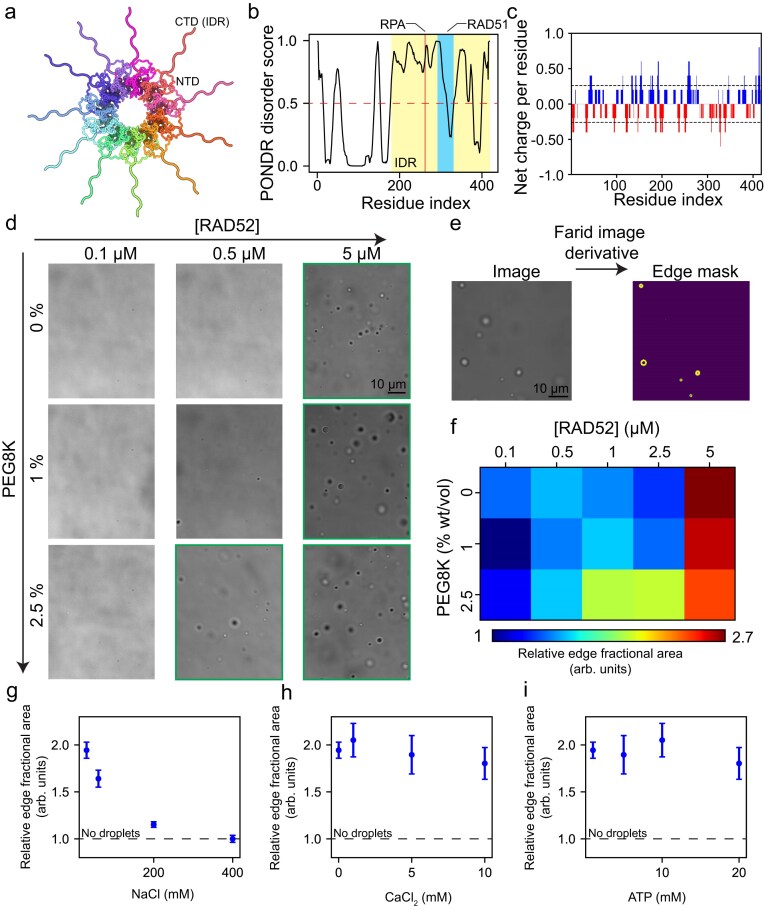
RAD52 undergoes homotypic phase separation. (**a**) A scheme showing the structure of the RAD52 undecameric ring visualized using Protein Imager [78] based on a previously reported cryo-EM structure [79] (PDB ID 8BJM). The IDRs are manually drawn to illustrate their presence and are not to scale. (**b**) PONDR (predictor of natural disordered regions) score of the RAD52 protein showing that its C-terminal half is highly disordered [80]. RAD51 and RPA binding regions are also annotated [62, 64]. (**c**) Net charge per residue as a function of residue index for RAD52 as calculated by the CIDER software from R. Pappu’s lab [81]. (**d**) Brightfield images of RAD52 mixtures under different protein and crowding conditions. (**e**) An example of Farid filter application to detect droplet edges in a brightfield image, which is used to quantify the degree of droplet formation in images (see “Materials and methods” section). (**f**) Quantitative phase separation state diagram of RAD52-PEG mixtures (see Supplementary Fig. S1). (**g**) RAD52 droplet formation as a function of sodium chloride. (**h**) RAD52 droplet formation as a function of calcium chloride. (**i**) RAD52 droplet formation as a function of ATP. In (g–i), the dashed line represents a non-phase separating sample. Additionally, these samples (g–i) were made in the absence of polymer crowders. Brightfield images are shown in Supplementary Fig. S3.

The human RAD52 protein forms an undecamer with a structured core ring surrounded by eleven disordered C-terminal regions (Fig. 1a and b). Analysis of the linear net charge per residue shows that the RAD52 polypeptide contains both positively and negatively charged segments (Fig. 1c). Accordingly, we hypothesized that phase separation may occur at low salt concentrations via Coulomb interactions between oppositely charged regions. To test this, we prepared RAD52 solutions in 30 mM NaCl concentration and varied the protein concentration from 0.1 to 5 µM. Under non-crowded conditions, phase separation was observed at 5 µM (Fig. 1d and f; Supplementary Fig. S1). We then introduced polyethylene glycol (PEG8000), a pseudo-inert polymer, to mimic intracellular crowding. PEG and other crowding agents reduce the threshold concentration for phase separation through the excluded volume effect [74]. Simply put, crowding agents occupy space in solution, thereby decreasing the available volume for proteins and promoting attractive interactions [74, 75]. Adding 1% wt/vol PEG reduced the condensation threshold to 1 µM (Fig. 1d), and further addition of PEG resulted in phase separation at concentrations as low as 250 nM (Fig. 1d). This low threshold is likely attributable to the large size of the undecameric complex (∼10 nm ring diameter [57]) and the unusually high multivalency of its eleven IDRs. Notably, the concentration of proteins and nucleic acids in the nucleus is estimated to be in the range of 100–200 mg/ml, corresponding to 10%–20% crowding conditions [76]. To quantify phase separation, we employed image analysis that detects droplet-dilute phase interfaces using a Farid filter [77] that calculates image derivatives (Fig. 1e, see “Materials and methods” section). A quantitative state diagram of PEG versus RAD52 concentration illustrates that phase separation is promoted in a crowder concentration-dependent manner (Fig. 1f). Additionally, to assess the role of the disordered C-terminal domain in phase separation, we tested a truncated RAD52 variant (AA: 1-212, RAD52ΔCTD). This variant did not undergo phase separation under the same experimental conditions (Supplementary Fig. S2), implicating the disordered domain as a key driver of phase separation.

We next tested the stability of RAD52 condensates against monovalent salts. Increasing NaCl suppressed phase separation, with droplet dissolution observed at around 200 mM NaCl (Fig. 1g and Supplementary Fig. S3a), confirming that Coulomb interactions between oppositely charged regions are the primary driving force. Finally, considering that *in vitro* recombination assays typically include divalent salts and ATP, we evaluated the effects of calcium and ATP on RAD52 phase separation. Under non-crowding conditions, neither calcium nor ATP (1–20 mM) significantly altered condensation behavior (Fig. 1 h and i; Supplementary Fig. S3b and c).

**Figure 2.**
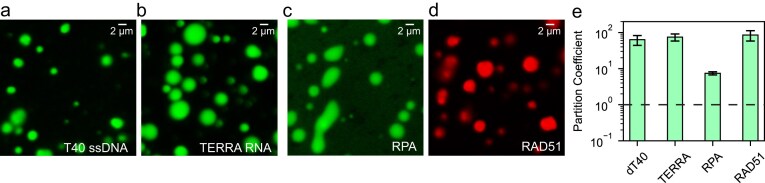
RAD52 condensates recruit DSB repair components. (**a**–**d**) Fluorescent images of RAD52 condensates (prepared at 5 µM RAD52) in the presence of dT40-Cy3, TERRA RNA-Cy3, RPA-MB543, and RAD51-Cy5, respectively. (**e**) Measured average partition coefficient of the client molecules (a–d) obtained from image analysis of multiple droplets per sample (*n*_condensates _> 100). Error bars represent one standard deviation. The dashed line represents a partition coefficient of 1, indicating no preferential interactions between the client molecule and the droplet.

Previous studies have shown that the structured ring formed by the N-terminal region of RAD52 contains two binding sites for ssDNA [82]. In addition, the C-terminal region of RAD52 interacts with RAD51 and RPA [64, 83], which are key factors in HR and other DNA repair pathways. Based on this, we tested whether RAD52 condensates can recruit and concentrate these biomolecules. We measured the partition coefficient (droplet intensity/dilute phase intensity) of fluorescently labeled 40-nt poly(T) ssDNA (hereafter referred to as dT40), TERRA [84], RPA, and RAD51 within RAD52 condensates. All four components were efficiently recruited by RAD52 condensates (Fig. 2a–d), with partition coefficients significantly >1 (Fig. 2e). This ability to concentrate DNA and repair proteins suggests that RAD52 condensation may contribute to the formation of RAD52 repair centers, as previously observed in cancer cells following DNA damage [26, 34].

### ssDNA and RPA regulate RAD52 condensation

In a cellular context, changes in expression levels and stoichiometries allow for concentration-dependent regulation of protein self-assembly [55, 85]. Previous studies have shown that heterotypic phase separation is typically governed by the stoichiometry of the interacting components [66, 86, 87]. Given that RAD52 contains binding domains for ssDNA and DNA repair proteins, we hypothesized that its phase separation may be modulated by the presence of these components in a concentration-dependent manner.

**Figure 3.**
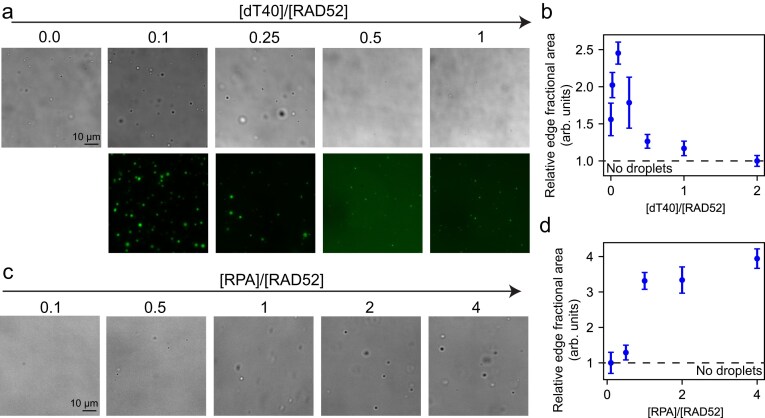
Regulation of RAD52 phase separation by ssDNA and RPA. (**a**) Brightfield and fluorescence images of RAD52–ssDNA mixtures at 2.5 µM RAD52 and variable dT40-to-RAD52 concentration ratio as indicated. dT40 in these experiments is labeled with Cy3 for fluorescence visualization. (**b**) Quantification of droplet formation via image analysis of the data in panel (**a**) and in Supplementary Fig. S4. Dashed line indicates the no-droplet condition at 1. (**c**) Brightfield images of RAD52–RPA mixtures prepared at 2.5 µM RAD52 and variable RPA concentration. (**d**) Quantification of the images in panel (**c**) via measuring the interface fractional area normalized to a non-phase separating sample. The dashed line indicates the no-droplet condition at 1.

To test this idea, we prepared RAD52 at 2.5 µM concentration in the absence of polymer crowders and varied the concentration of ssDNA dT40 (Fig. 3a and b; Supplementary Fig. S4). Notably, the length of dT40 matches the circumference of the RAD52 ring [62]. At low molar fraction ([dT40]/ [RAD52]∼0.1), corresponding to one ssDNA molecule per RAD52 undecamer, we observed enhanced condensate formation (Fig. 3a and b; Supplementary Fig. S4). However, at higher ssDNA-to-RAD52 ratios (>0.5), phase separation was completely suppressed (Fig. 3a and b; Supplementary Fig. S4). This non-monotonic behavior, known as a reentrant phase transition (where the system reverts to a homogeneous solution [66, 88]), has been previously observed in mixtures of cationic proteins and nucleic acids, and is considered a general feature of heterotypic phase-separating systems [66, 86, 89, 90].

We next investigated the influence of RPA, a known RAD52 interaction partner. RPA enhanced phase separation at low RPA-to-RAD52 mixing ratios (Fig. 3c and d). Unlike ssDNA, no reduction in phase separation was observed at high RPA concentrations, suggesting that the decondensation threshold may lie beyond the concentration range explored. In control experiments omitting RAD52, phase separation was not observed for RPA or ssDNA (Supplementary Fig. S5).

We next examined whether RAD52–ssDNA and RAD52–RPA condensates can form compatible phases. Previous studies showed that multicomponent mixtures can give rise to either a single mixed condensed phase or multiple condensed phases that remain demixed [91]. The emergence of coexisting phases with distinct compositions is typically governed by the hierarchy of interaction networks [92].

To assess compatibility, we generated RAD52–ssDNA and RAD52–RPA condensates separately (labeled with 18-mer Cy5-conjugated ssDNA and RPA-MB543, respectively, see “Materials and methods” section), and then mixed them prior to imaging. Two-color fluorescence microscopy revealed that most condensates contained both RPA and RAD52 (Supplementary Fig. S6), indicating the formation of tri-component condensates composed of RAD52, RPA, and ssDNA. Together, these findings demonstrate that RAD52 phase separation is strongly influenced by RPA and ssDNA in a concentration-dependent manner. Importantly, RAD52 condensation is regulated non-monotonically by ssDNA through a reentrant phase transition, a phenomenon that may represent a key molecular mechanism for controlling condensation behavior *in vivo* [93]. Cellular conditions that alter intracellular component concentrations, such as RAD52 overexpression, accumulation of ssDNA through end resection, or elevated RPA levels following extensive DNA damage, may promote RAD52 phase separation. Indeed, RAD52 foci formation has been observed in BRCA2- or BRCA1-deficient cells upon DNA damage [53]. We speculate that altered intracellular stoichiometry may play a critical role in regulating RAD52 condensation dynamics [18].

### RAD52 induces RAD51–ssDNA fibril formation

**Figure 4.**
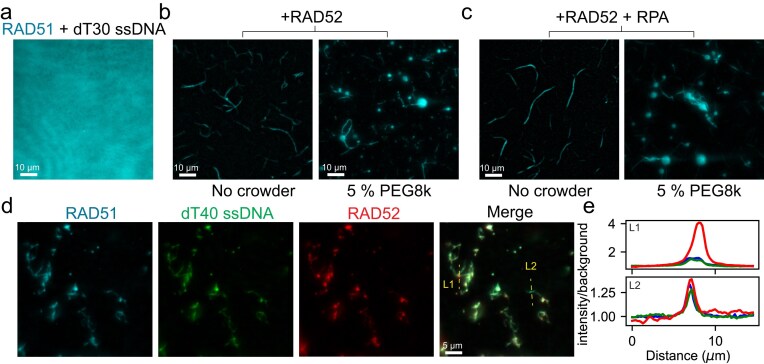
RAD52 induces RAD51–ssDNA fibril formation. (**a**) Fluorescent image of RAD51–ssDNA solution prepared at 5 µM RAD51 and 2.5 µM ssDNA dT30. Also added to the sample is ∼200 nM of AF488-conjugated RAD51. (**b**) Fluorescent images of the same sample in panel (a) but with an additional 5 µM of RAD52 in the presence and absence of a polymer crowder (PEG8k). (**c**) Fluorescent images of the same sample in panel (b) but with the addition of 2.5 µM RPA. (**d**) Three-color fluorescent images of RAD51–RAD52–dT40 sample (5, 5, and 2.5 µM, respectively) at no-crowder condition. The sample also contained ∼200 nM of Cy5-conjugated RAD52 and AF488-conjugated RAD51. (**e**) Intensity profile for the fluorescence signal across the two yellow lines in panel (d) for RAD51 (blue), dT40 (green), and RAD52 (red). Line L1 is drawn across a spherical object that concentrates RAD52 more than RAD51 and the ssDNA. Line L2 is drawn across a fiber that contains all three components (RAD51, RAD52, and ssDNA) in similar ratios.

HR relies on the assembly of the RAD51 NPF on resected ssDNA overhangs to initiate homology search. This assembly is facilitated by BRCA2, which promotes the replacement of RPA with RAD51 on ssDNA [19, 94]. In cells lacking functional BRCA2, NPF formation is believed to proceed via an alternative pathway. To test whether RAD52 phase separation influences the interactions between RAD51 and ssDNA, we prepared mixtures containing 5 µM RAD51 (including 10% RAD51-AF488) and 2.5 µM ssDNA (dT30) in a buffer devoid of ATP and divalent ions. Under these conditions, no detectable structures were observed (Fig. 4a). However, addition of 5 µM RAD52 to the same mixture induced the formation of elongated, fiber-like RAD51 structures exceeding 10 µm in length (Fig. 4b). Adding a crowding agent (5% PEG8K) promoted the formation of condensates that colocalize with and recruit RAD51 along the RAD51 fibers (Fig. 4b). Remarkably, RAD51 fiber formation persisted even in the presence of RPA at equimolar concentration to dT30 (2.5 µM, Fig. 4c), despite RPA’s known ability to block RAD51–ssDNA interactions [67]. Three-color fluorescence imaging of RAD51, RAD52, and ssDNA confirmed that these fibrillar structures contain all three components. In addition, spherical condensate-like structures were observed to sequester large amounts of RAD52, as evidenced by its high partitioning relative to RAD51 and ssDNA (Fig. 4d and e; Supplementary Fig. S7). Interestingly, when the mixing order was altered such that RAD52 is added prior to RAD51, fibril formation was not observed (Supplementary Fig. S8), suggesting that fibrilization occurs specifically through interactions between RAD52 and preassembled RAD51–ssDNA complexes. Furthermore, fibril formation was confirmed in the presence of ATP and calcium, conditions necessary for RAD51 recombinase activation (Supplementary Fig. S9). Taken together, these results demonstrate that RAD52, irrespective of its phase separation, promotes the formation of RAD51–ssDNA fibrils when added post-complex formation. Molecular crowding further contributes to the formation of RAD52 condensates that recruit RAD51 and ssDNA, albeit to a lesser degree.

### Visualization of individual RAD51 nucleoprotein filaments using DNA-PAINT

Visualization of individual RAD51 NPFs and their interactions with repair proteins could offer crucial insights into the mechanisms underlying RAD51 fibril formation described earlier. High-resolution imaging of RAD51 NPFs has historically relied on AFM and electron microscopy (EM) [95, 96]; however, both techniques are label-free and cannot readily distinguish among different protein components. Single-molecule FRET experiments have demonstrated that RAD51 filament formation extends DNA substrate length [62], while confocal microscopy has visualized RAD51 filaments on dsDNA, though with diffraction-limited resolution [97]. Cryo-EM has also been employed to resolve RAD51 NPF structures [98], but imaging aggregated or clustered filaments remains problematic due to population heterogeneity and the large size of these supramolecular assemblies. Moreover, functional assays evaluating RAD51 NPF-mediated strand invasion of homologous dsDNA have generally relied on gel-based assays or EM imaging [7, 19].

In this section, we developed a super-resolution imaging approach capable of probing RAD51 NPF structure and its interactions with RAD52, RPA, and homologous DNA substrates. To this end, we applied DNA-PAINT [71] to visualize RAD51 filaments, D-loops, and filament clustering at ∼10 nm resolution using the large DNA substrate of $\phi $X174 virion DNA. The $\phi $X174 bacteriophage genome is a 5.8 kb ssDNA, commercially available along with its dsDNA form.

**Figure 5.**
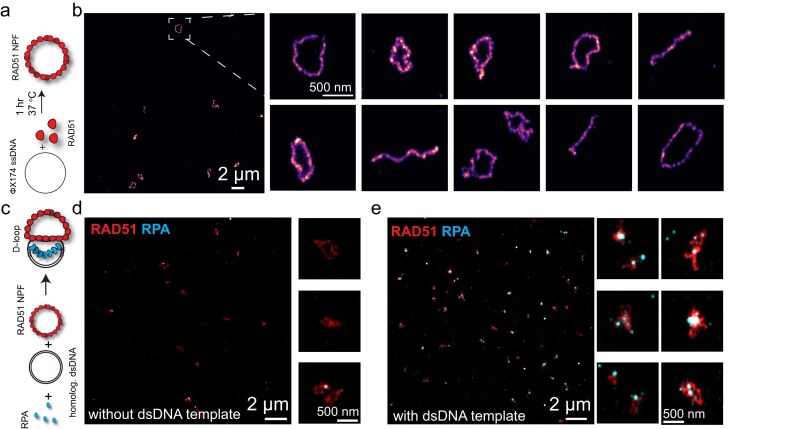
Super-resolution imaging to visualize RAD51 NPFs. (**a**) A scheme showing the assembly reaction of RAD51 NPF. (**b**) A representative large view of RAD51 NPFs on a coverslip surface reconstructed from DNA-PAINT imaging. Also shown are zoomed-in views of individual RAD51 NPFs. (**c**) A scheme showing the D-loop formation reaction in the presence of a homologous dsDNA template. (**d**) Two-color DNA-PAINT image of RAD51 NPFs (red) and RPA (cyan) in the absence of dsDNA template. Also shown are zoomed-in views of individual RAD51 NPFs. (**e**) Two-color DNA-PAINT image of RAD51 (red) and RPA (cyan) in the presence of a dsDNA template. The colocalization of the two proteins indicates the formation of D-loops. Also shown are zoomed-in views of individual D-loop structures.

To assemble RAD51 NPFs, we mixed RAD51 with ssDNA under optimized conditions, including appropriate concentrations of monovalent and divalent salts and ATP (see “Materials and methods” section), and maintained the mixture at 37°C for 1 h (Fig. 5a). Complexes were then deposited on a poly-L-lysine-coated coverslip for subsequent immunostaining. Primary antibodies against RAD51 were followed by fragment secondary antibodies conjugated to oligonucleotide probes for DNA-PAINT (see “Materials and methods” section). Fluorescent imager strands complementary to the probe oligos were added at appropriate concentrations to enable rapid blinking for image reconstruction. Imaging was performed on an objective-type TIRF microscope. Our approach enabled the resolution of single NPF complexes at ∼15 nm (Fig. 5b and Supplementary Fig. S10). We observed multiple circular RAD51 filaments within a single field of view, along with several linear filament structures (Fig. 5b).

We then asked whether our super-resolution approach could detect D-loop formation. Pre-assembled NPFs on $\phi $X174 ssDNA were incubated with homologous dsDNA ($\phi $X174 RF I), and RPA was used to stain the displaced ssDNA portion within the D-loop structure (Fig. 5c). We expected colocalization between RPA and RAD51 to indicate D-loop formation, although full contour resolution was not anticipated due to the high flexibility of RPA-bound ssDNA. Indeed, NPFs assembled without dsDNA showed little to no RPA staining (Fig. 5d), whereas NPFs incubated with dsDNA revealed strong colocalization between RAD51 and RPA (Fig. 5e), supporting the formation of D-loops as illustrated in Fig. 5c. It is worth noting that the DNA-PAINT super resolution imaging of RAD51 NPFs appeared less efficient in Fig. 5d (as compared to Fig. 5b) potentially due to antibody crosstalk or increased background due to the presence of two different imager strands.

### RAD52 induces clustering and end-to-end tethering of RAD51 nucleoprotein filaments

**Figure 6.**
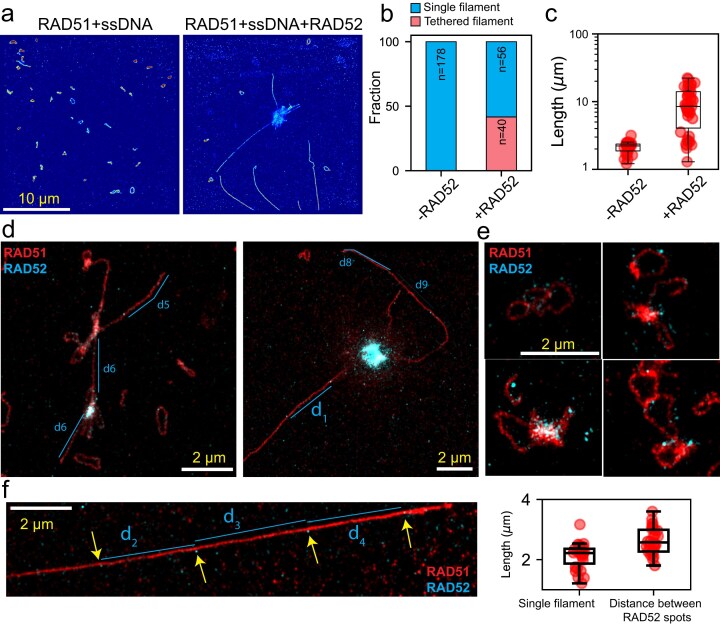
RAD52 induces RAD51 NPF clustering and end-to-end tethering. (**a**) DNA-PAINT super-resolution image of RAD51 NPFs formed on $\phi $× 174 ssDNA virion in the absence of RAD52 (left) and the presence of 0.5 µM RAD52. (**b**) Quantification of the single filaments and the tethered filaments in panel panel (a) was obtained from analyzing multiple images. (**c**) DNA-PAINT image of RAD52-induced clustering of RAD51 NPF assembled on dsDNA. (**d**) Two representative DNA-PAINT images of RAD52 (cyan) and RAD51 NPF (red) clusters showing elongated RAD51 NPFs that are likely to be tethered (also see Supplementary Fig. S13). (**e**) Two-color images of clustered circular RAD51 NPFs (red) in the presence of RAD52 (cyan). (**f**) Example of an excessively long RAD51 NPF with equally spaced localizations of RAD52 (cyan, yellow arrows). The box plot on the right shows the measured distance between RAD52 spots in the left image (**d**) and Supplementary Fig. S13. The RAD52 inter-spot distance is compared with the measured length of single RAD51 NPFs from the data in panel (a). Note that the blue lines are drawn as a guide to the eye. The inter-spot distance is measured manually but calculating the number of pixels between two consecutive bright spots or junctions along a RAD51 filament.

To investigate interactions between RAD52 and RAD51 NPFs, we carried out the RAD51 NPF assembly reaction with and without RAD52 and conducted super-resolution imaging with DNA-PAINT. All samples contained 1 µM RPA to ensure a structure-free DNA template. In the absence of RAD52, RAD51 NPFs appeared uniformly distributed on the coverslip surface and consistently formed single, unclustered filaments (Fig. 6a, left). In contrast, the addition of RAD52 induced pronounced clustering of RAD51 NPFs (Fig. 6a, right). This clustering likely arises from RAD52 acting as a hub that attracts individual filaments via its multiple IDRs. We also observed extended RAD51 NPFs emanating from these clusters, with ∼40% of detected filaments exhibiting lengths significantly greater than those formed without RAD52 (Fig. 6a, right; Fig. 6b). Specifically, filament lengths increased from 2.1 ± 0.4 µm without RAD52 to 9 ± 6 µm with RAD52 (Fig. 6c). Importantly, these extended filaments were detected across multiple RPA concentrations (Supplementary Fig. S11) and were also present when filaments were assembled on the dsDNA substrate, albeit with overall shorter lengths (Supplementary Fig. S12). We hypothesized that these long filaments may be individual RAD51–DNA complexes tethered together by RAD52. To test this, we performed two-color DNA-PAINT imaging to visualize RAD52 within the clusters. Indeed, RAD52 was found colocalized with clustered RAD51 filaments (Fig. 6d). We also observed instances where multiple curved filaments are connected at discrete attachment points containing RAD52 localizations (Fig. 6e). Notably, some RAD52 localizations appeared evenly spaced along linear RAD51 filaments (Fig. 6d and f; Supplementary Fig. S13). The spacing between these RAD52 puncta was slightly greater than the measured length of individual RAD51 filaments formed on ssDNA, potentially due to filament stretching or limitations in tracing circular filament geometry (Fig. 6f and Supplementary Fig. S13). Although faint RAD52 localizations were scattered across filaments, the repeated appearance of bright, evenly spaced RAD52 puncta suggests that RAD52 rings may tether RAD51-DNA filaments in an end-to-end manner.

Together, these observations reveal two distinct modes of RAD52 interaction with RAD51 filaments: (i) segment binding, where RAD52 bridges multiple filaments laterally (Fig. 6d and e), and (ii) end-binding, where RAD52 links filaments longitudinally (Fig. 6d and f; Supplementary Fig. S13). Whether RAD52 favors one mode over the other remains unclear. Previous studies have suggested that yeast Rad52 preferentially binds to ssDNA ends [99], and RAD52 has been proposed to recognize ssDNA–dsDNA junctions in the presence of RPA [29]. In summary, the combination of segment- and end-binding interactions likely accounts for the formation of long RAD51–ssDNA fibrils observed in this work.

**Figure 7.**
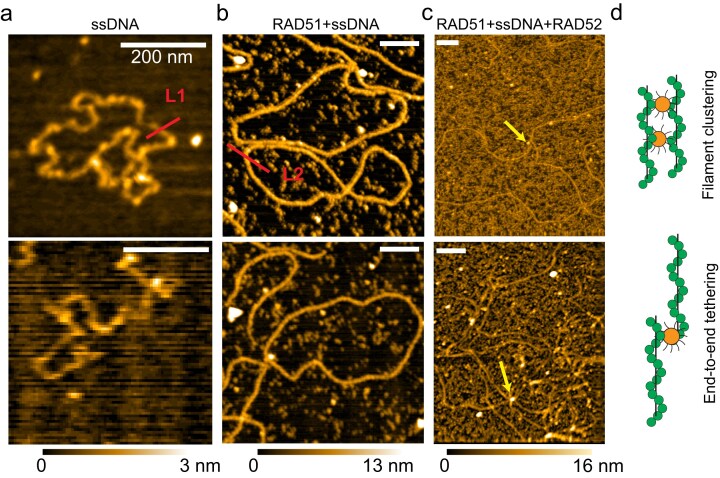
AFM confirms RAD52-induced clustering and tethering of RAD51 NPFs. (**a**) AFM images of naked $\phi $× 174 ssDNA in its circular (top) and linear (bottom) forms. (**b**) AFM images of RAD51 NPF. (**c**) AFM image of RAD51 NPFs in the presence of 0.5 µM of RAD52. All scale bars are 200 nm. Arrows indicate a molecule that is likely to be RAD52 crosslinking filaments. (**d**) Scheme showing the two bridging modes of RAD52 that can either cluster or tether RAD51 filaments.

To further validate our findings from super-resolution imaging, we employed AFM to image RAD51 NPFs and RAD52-RAD51 complexes without the use of fluorophore labeling or antibody staining. We first imaged the ssDNA template and confirmed the presence of both circular and linear chains (Fig. 7a and Supplementary Fig. S14). Next, we examined pre-assembled RAD51 filaments, confirming that these filaments were spatially separated and did not exhibit inter-filament clustering (Fig. 7b and Supplementary Fig. S14). When RAD51 NPFs were assembled in the presence of RAD52 and RPA, we observed large-scale filaments that were extensively tangled and extended beyond the field of view (4 × 4 µm for our AFM setup, Fig. 7c and Supplementary Fig. S15). These long filaments were interspersed with large blobs that we identify as RAD52, since RAD52 rings are ∼10 nm in diameter and are much larger than RAD51. We also observed networks of multiple elongated RAD51 filaments seemingly crosslinked by RAD52 blobs (Fig. 7c, bottom). The increased background in Fig. 7c is attributable to the higher RPA concentration (1 µM), which was necessary to ensure the presence of unstructured ssDNA and facilitate the formation of extended filaments. We note that the increased RPA concentration is to offset the potential competition between RAD52 and RPA binding to ssDNA and also to ensure all possible modes of RPA binding. The RAD51 extended filaments were also observed at lower RPA concentrations (see Supplementary Fig. S11). In sum, our AFM imaging results corroborate our findings from DNA-PAINT and reveal a previously unappreciated ability of RAD52 to drive clustering and end-to-end tethering of RAD51 NPFs, culminating in the formation of extended RAD51 filament assemblies (Fig. 7d).

## Discussion

The human RAD52 complex holds considerable potential for cancer therapy, as its disruption is selectively toxic to cells lacking a functional BRCA2 gene [85]. Loss of BRCA2 underlies multiple cancer types and development disorders, collectively known as HR deficiency syndrome [100]. Extensive research has elucidated the biochemical activities of RAD52, including ssDNA annealing and reverse strand exchange reactions [41, 52], as well as its interaction with ssDNA. However, the self-assembly properties of the human RAD52 and its interaction with RAD51 NPFs have not been fully documented. Here, we address this gap by investigating RAD52’s ability to self-assemble and engage RAD51 NPFs. We demonstrate that RAD52 undergoes phase separation at sub-micromolar concentrations in crowded environments. These RAD52 condensates recruit nucleic acids and DSB repair, suggesting that they model the RAD52 repair centers previously reported. Moreover, RAD52 promotes the formation of RAD51–ssDNA fibril-like structures that span tens of microns. Single-molecule imaging revealed RAD52-mediated clustering and end-to-end tethering of linear RAD51 NPFs. Collectively, our data unveil unique organizational behaviors of the RAD52 complex, likely arising from its branched multivalent architecture.

The phase separation properties of RAD52 may contribute to the formation of RAD52 foci in cells that act as DSB repair hubs that recruit RAD51 and ssDNA [101]. This supports an emerging paradigm connecting phase separation with cellular DNA damage responses [102]. Notably, yeast Rad52 was recently shown to phase separate in the nucleus and induce nuclear microtubule formation [103]. RAD51 itself was found to form amyloid fibrils at high concentrations [104], and two independent studies reported ∼1 µm-thick RAD51 fibril-like structures in human cells upon RAD51 overexpression [104, 105]. These structures [105] resemble the RAD52-induced fibrils shown in Fig. 4. Interestingly, the TR2 domain of BRCA2, which is a RAD51 binding domain, induces bundling of RAD51-ssDNA NPFs in vitro [94]. This specific domain is required for the BRCA2 function in protecting stalled replication forks [106]. Our findings echo this behavior, showing RAD52-mediated bundling of RAD51–ssDNA complexes. RAD52’s known role in stalled fork protection [43] further supports this parallel. Analogous bundling has been observed for RecA, a bacterial homolog of RAD51, upon DNA damage [107]. These RecA bundles, comprising both DNA-bound and DNA-free filaments, are thought to facilitate ssDNA transport along the RecA fiber via sliding, enabling homology pairing. The human RAD51 fibers generated under RAD52 influence resemble these RecA structures [107] as well as those formed in cells overexpressing RAD51 [105]. Further investigation is required to define the physiological relevance of these RAD51 fibrils and their contribution to later HR stages, such as homology search.

Our single-molecule imaging revealed RAD52’s capacity to generate exceptionally long RAD51 filaments. Strikingly, RAD52 formed regularly spaced hubs along these extended filaments, with inter-blob spacing approximating the length of individual RAD51 NPFs. While previous reports describe mixed RAD51–RAD52 filaments [29], such heterotypic assemblies do not explain the extensive filament lengths observed, reaching tens of microns. Instead, we propose that RAD52 promotes end-to-end tethering of pre-formed RAD51 NPFs, a behavior consistent with its DNA end binding preference [108], reminiscent of the Ku heterodimer in the NHEJ pathway [109]. We speculate that this property may contribute to RAD52’s role in second-end capture during HR [110, 111]. When a DSB occurs, two RAD51 filaments form on the two ends of the DSB. After strand invasion, the ssDNA portion of the D-loop can engage the opposing RAD51 filament to form a joint protein–DNA complex that bridges the two ends of complementary strands of the same donor dsDNA. This phenomenon is known as second-end capture and is promoted by RAD52 [110, 111]. We argue that the ability of RAD52 to tether RAD51 NPF ends might contribute to its capacity to promote second-end capture by ensuring both DSB-induced RAD51 NPF remain together physically during homology search. Overall, our results add to the list of other observations on the mechanisms of action of RAD52 in stimulating strand invasion [27], aiding homology search [29], forming DNA networks via bridging multiple DNA strands [96], and ssDNA binding and wrapping [62, 112]. Together, these reports pave the way for a holistic understanding of the properties and functions of the RAD52 protein.

In conclusion, we have investigated the self-assembly properties of the human RAD52 complex and its interactions with DSB repair proteins and nucleic acids. We show that RAD52 can undergo phase separation, induce RAD51–ssDNA fibril formation, and facilitate RAD51 NPF clustering and end-to-end tethering. These properties may provide a mechanistic basis for the protein function in the cell, which may ultimately lead to advancing our current strategies of inhibiting RAD52 that are harnessed in drug-based cancer therapy.

## Supplementary Material

gkag043_Supplemental_File

## Data Availability

All the data pertaining to the conclusions of this manuscript are presented in the main text and supplementary information. Additional data are available upon reasonable request.
