# Supplementary material for: The human RAD52 complex undergoes phase separation and facilitates bundling and end-to-end tethering of RAD51 presynaptic filaments
Source: Nucleic Acids Res. 2026 Jan 28;54(3):gkag043. doi: 10.1093/nar/gkag043 (PMC12848948; doi:10.1093/nar/gkag043)
Supplement: gkag043_Supplemental_File [file gkag043_supplemental_file.pdf]

Supplementary information for

# The human RAD52 complex undergoes phase separation and facilitates bundling and end-to-end tethering of RAD51 presynaptic filaments

Ibraheem Alshareedah[a,b], Sushil Pangeni[a,c], Paul A. Dewan Jr.[a,d], Masayoshi Honda[e], Ting-Wei Liao[a,c], Maria Spies[e], and Taekjip Ha[a,b,c*]

[a] Howard Hughes Medical Institute and Program in Cellular and Molecular Medicine, Boston Children's Hospital, Boston, MA 02115, USA

[b]Department of Pediatrics, Harvard Medical School, Boston, MA 02115, USA

[c]Department of Biophysics, Johns Hopkins University, Baltimore, MD 21205, USA

[d]Harvard Biophysics Graduate Program, Harvard University, Cambridge, MA, USA

[e]Department of Biochemistry and Molecular Biology, University of Iowa Carver College of Medicine, 51 Newton Road, Iowa City, IA 52242, USA

*Corresponding Author: Taekjip.Ha@childrens.harvard.edu

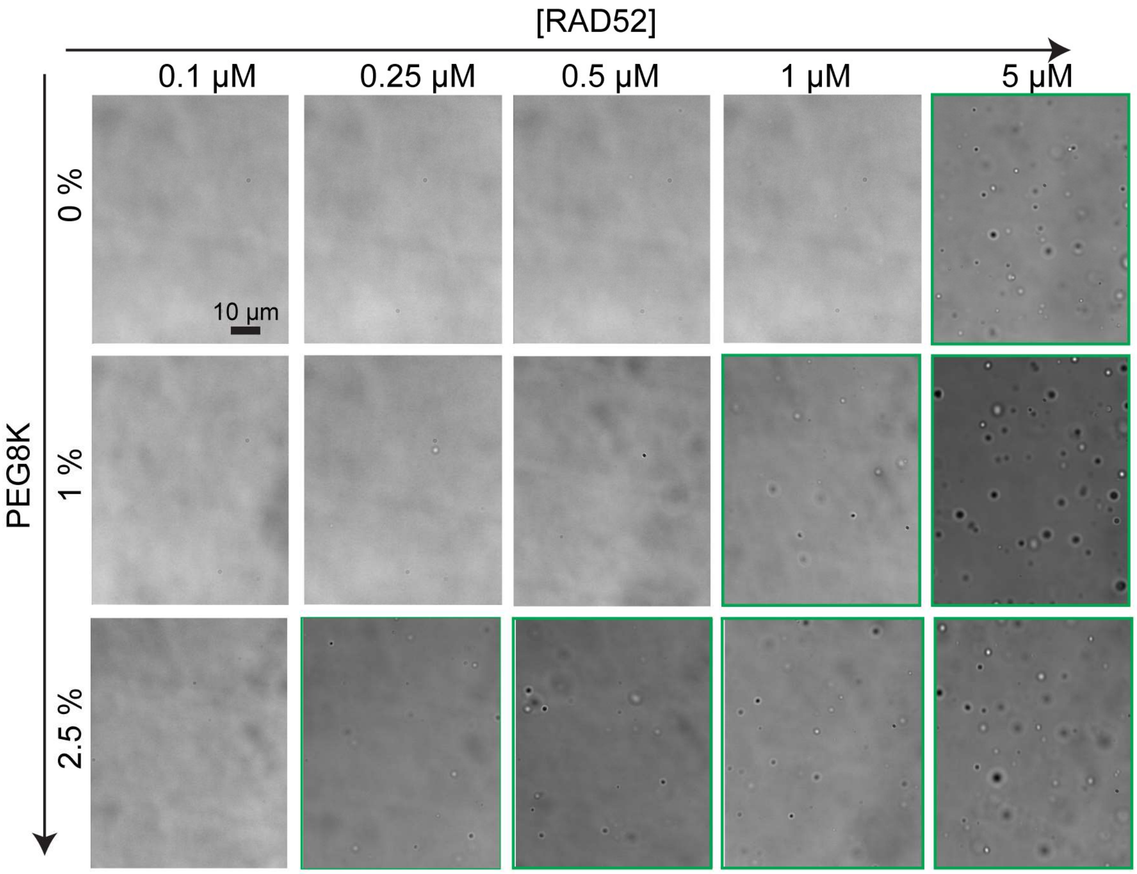


**Figure S1.** Bright-field images of RAD52 mixtures as a function of RAD52 concentration and PEG 8000 concentration (wt/vol). Scale bar is 10 μm. This is the complete set of conditions tested to compute the phase diagram in Figure 1f in the main text.

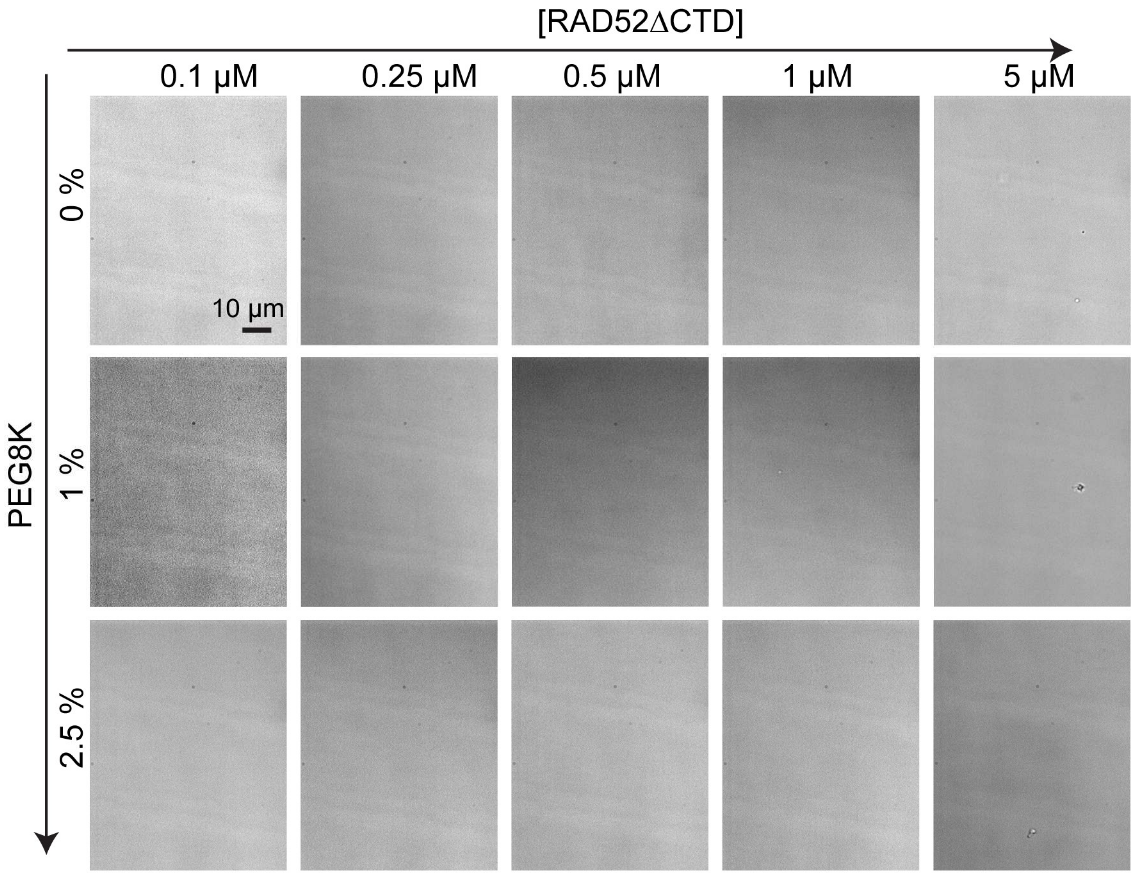


**Figure S2.** Bright-field images of RAD52ΔCTD mixtures as a function of RAD52ΔCTD concentration and PEG 8000 concentration (wt/vol). Scale bar is 10 μm.

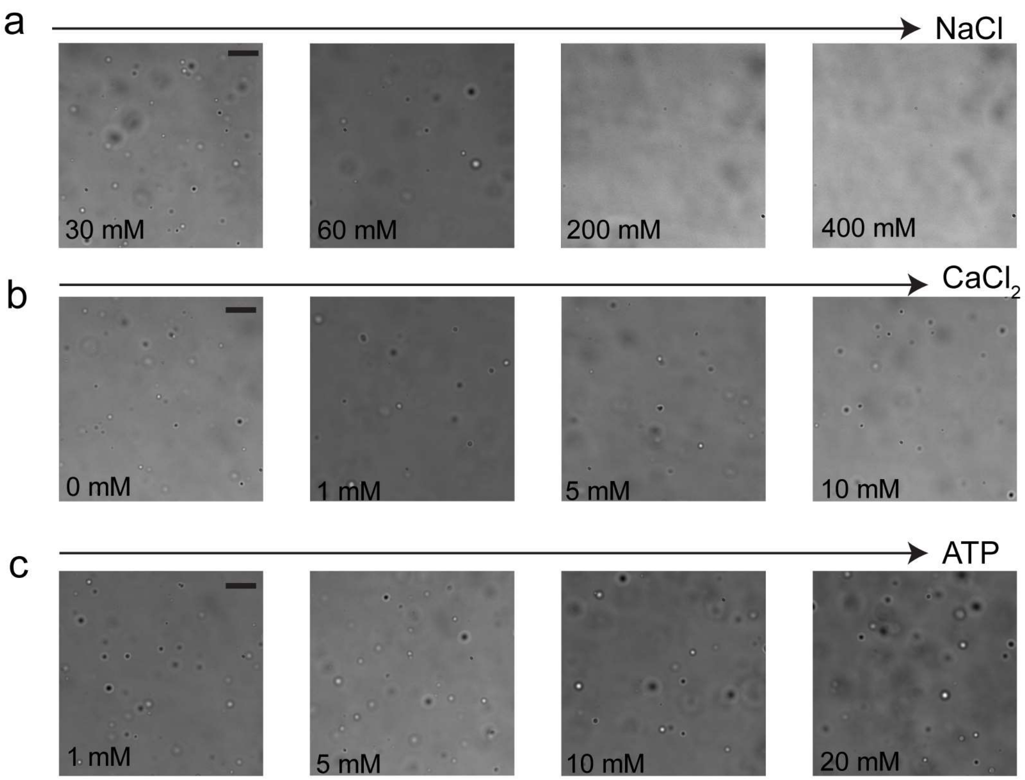


**Figure S3.** Bright-field images of RAD52 mixtures prepared at 5 μM RAD52 concentration and no crowder as a function of **(a)** NaCl, **(b)** $CaCl_2$, and **(c)** ATP concentrations. Scale bar is 10 μm. This is the complete set of conditions tested to compute the plots in Figure 1g-i in the main text.

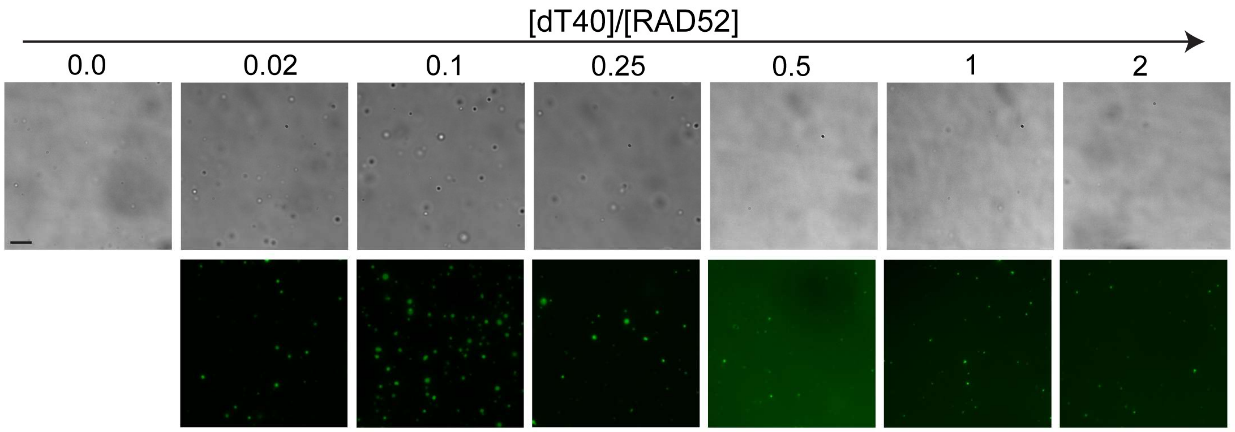


**Figure S4.** Bright-field (top) and fluorescence (bottom) images of RAD52-ssDNA mixtures prepared at 2.5 μM RAD52 concentration and variable ssDNA-to-protein ratio. Scale bar is 10 μm. This is the complete set of conditions tested that were used to compute the phase diagram plot in Figure 3b in the main text. dT40 ssDNA is conjugated to Cy3.

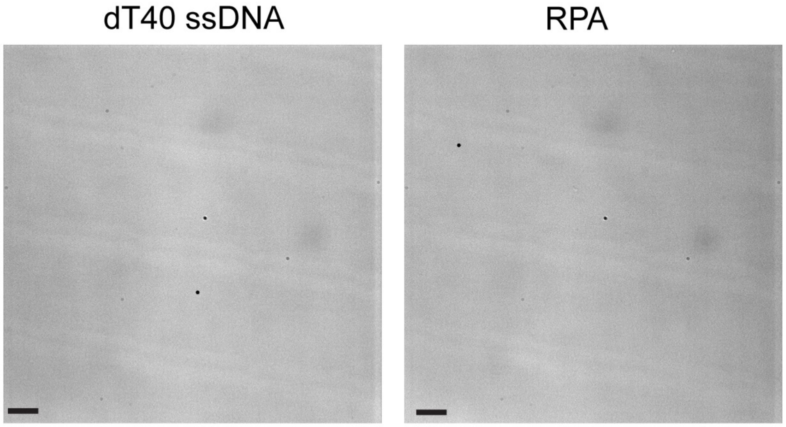


**Figure S5.** Bright-field images of DNA (dT40 at 0.625 µM) and RPA (4 µM with 1% PEG8k) samples formed at the concentration of maximum phase separation (see Figure 3) but without RAD52. These samples show no signs of phase separation, confirming that RAD52 is the trigger of phase separation observed in Figure 3 of the main text.

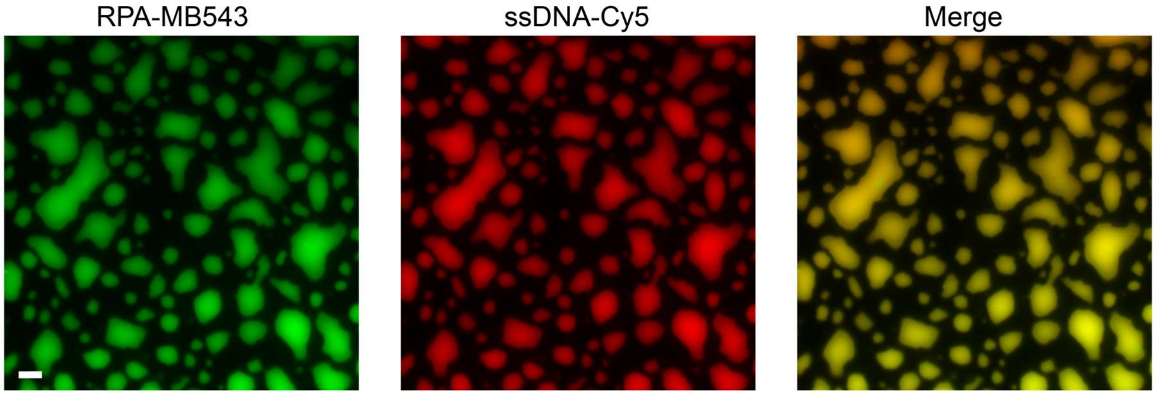


**Figure S6.** Multi-color fluorescence image of RAD52-dT40 condensates (with 200 nM Cy5-conjugated 18mer DNA probe) and RAD52-RPA (with 300 nM RPA-MB543) condensates mixed into one sample. Scale bar is 10 μm.

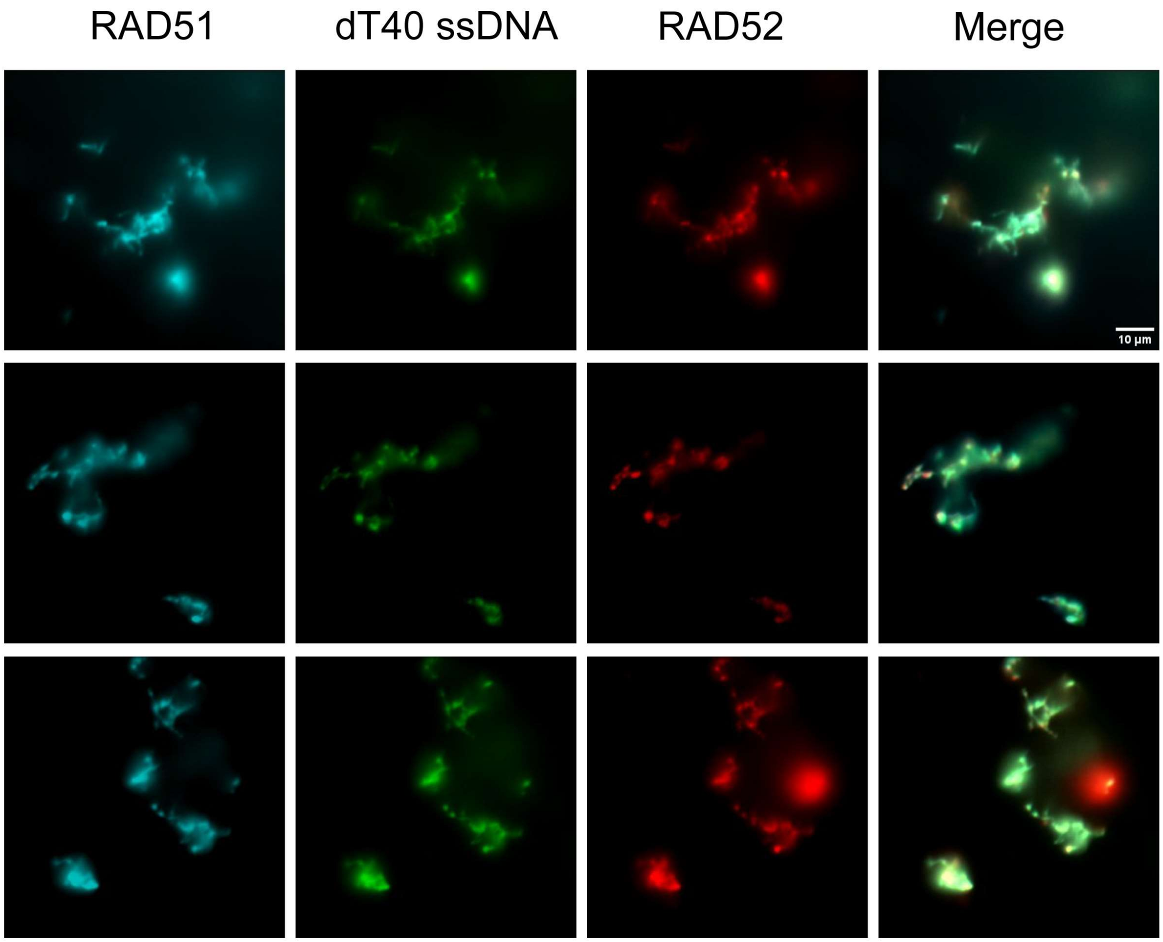


**Figure S7.** Multi-color fluorescence images of structures formed by a RAD51-RAD52-dT40 mixture (5 µM, 5 µM, 2.5 µM, respectively) at no crowder condition. This is the same sample in Figure 4d, with more examples of the complex structures formed by RAD51 fibrils and RAD52.

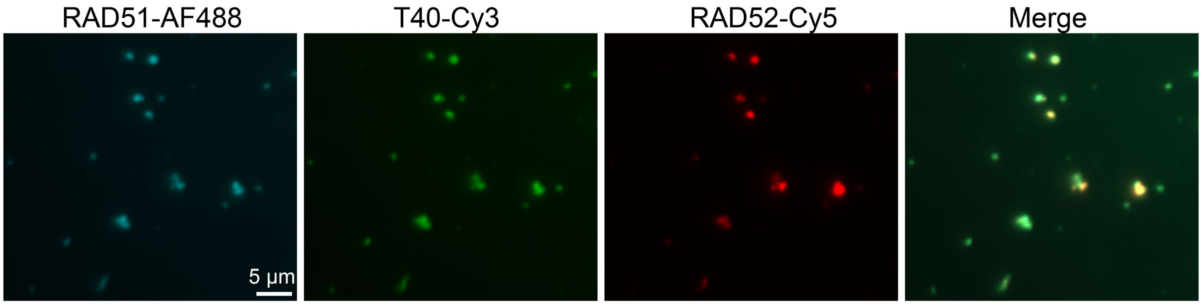


**Figure S8.** Multi-color fluorescence image of a RAD51-RAD52-dT40 mixture (5 µM, 5 µM, 2.5 µM, respectively) at no crowder condition. This sample is identical to the one shown in Figure 4d in the main text, however, the two samples differ in the order of addition. In this sample, ssDNA is added first followed by RAD52 and then RAD51. In Figure 4d, ssDNA is added first followed by RAD51 and then RAD52.

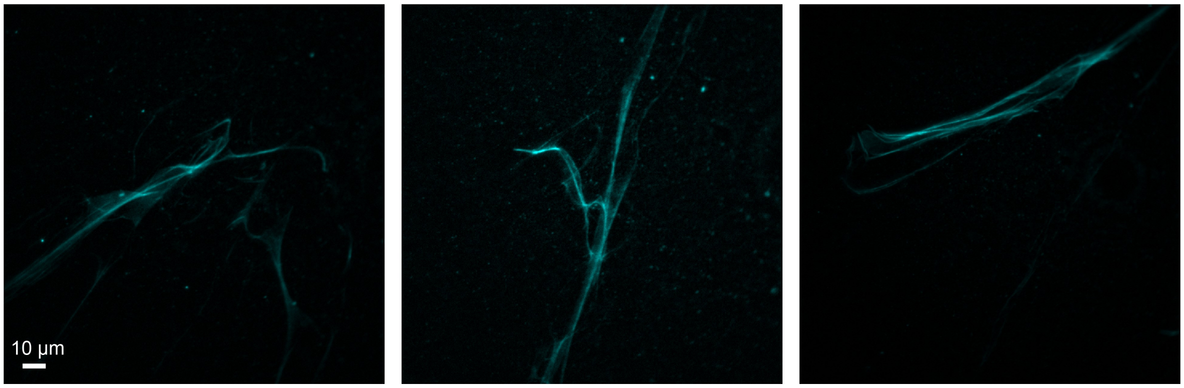


**Figure S9.** Fluorescence images of RAD51 (with 5% AF488-RAD51) fibers prepared by mixing RAD51 (5 μM) with ssDNA T30 (2.5 μM) and RAD52 (5 μM) in a buffer containing 25 mM Tris-HCl (pH 7.5), 100 mM NaCl, 2 mM ATP, and 2 mM $CaCl_2$.

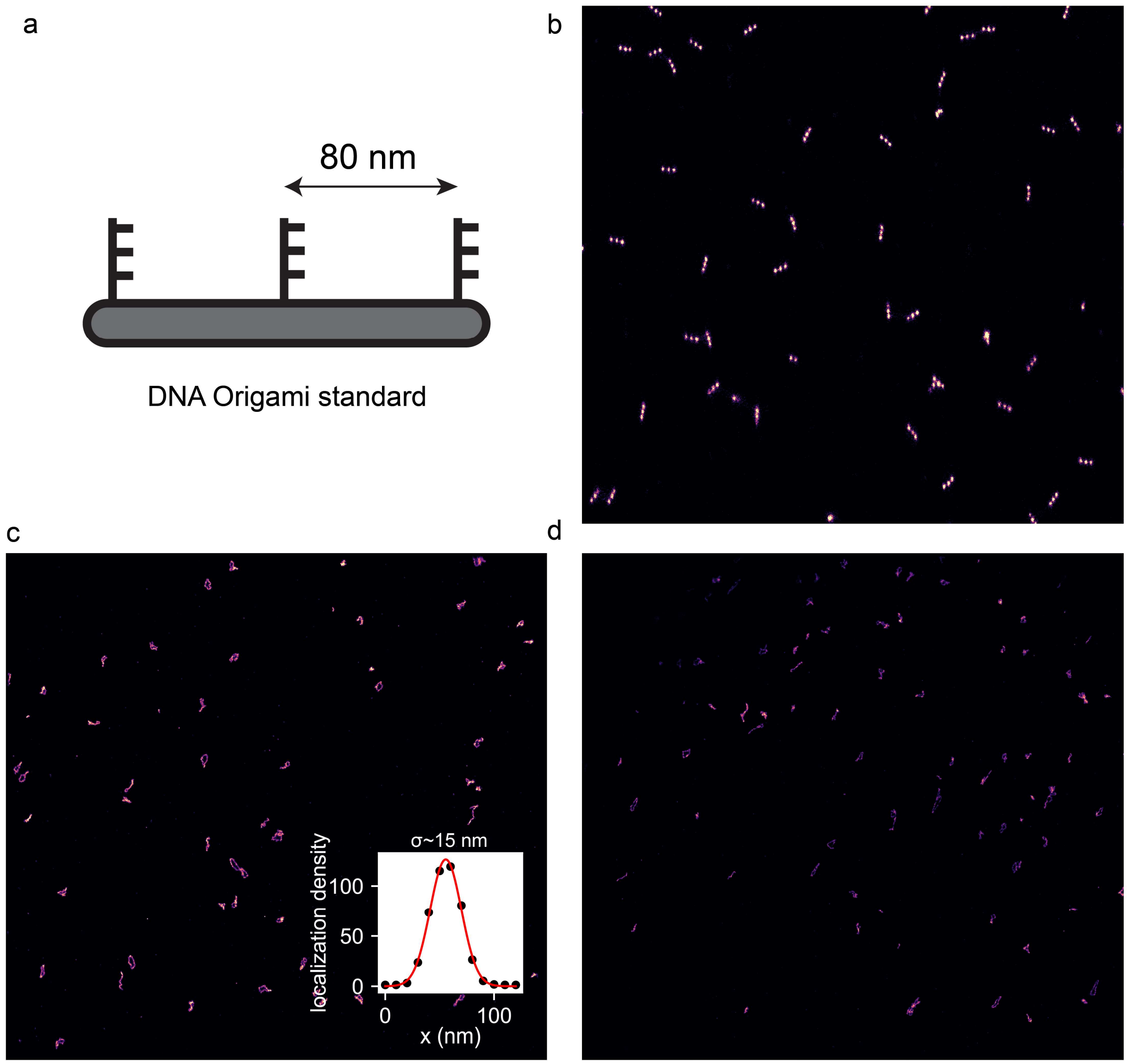


**Figure S10. (a)** Scheme describing a DNA origami nano-ruler (GATAquant) with probe strands located 80 nm apart that is used to judge the microscope resolution. **(b)** DNA paint image of the DNA nano-ruler **(c&d)** large view of the RAD51 NPF sample showing the circular and linear RAD51 NPFs formed on $\phi$X174 ssDNA. The standard deviation of the gaussian fit for a single protein localization is shown in the inset of **(c)** which gives an estimate of 15 nm resolution.

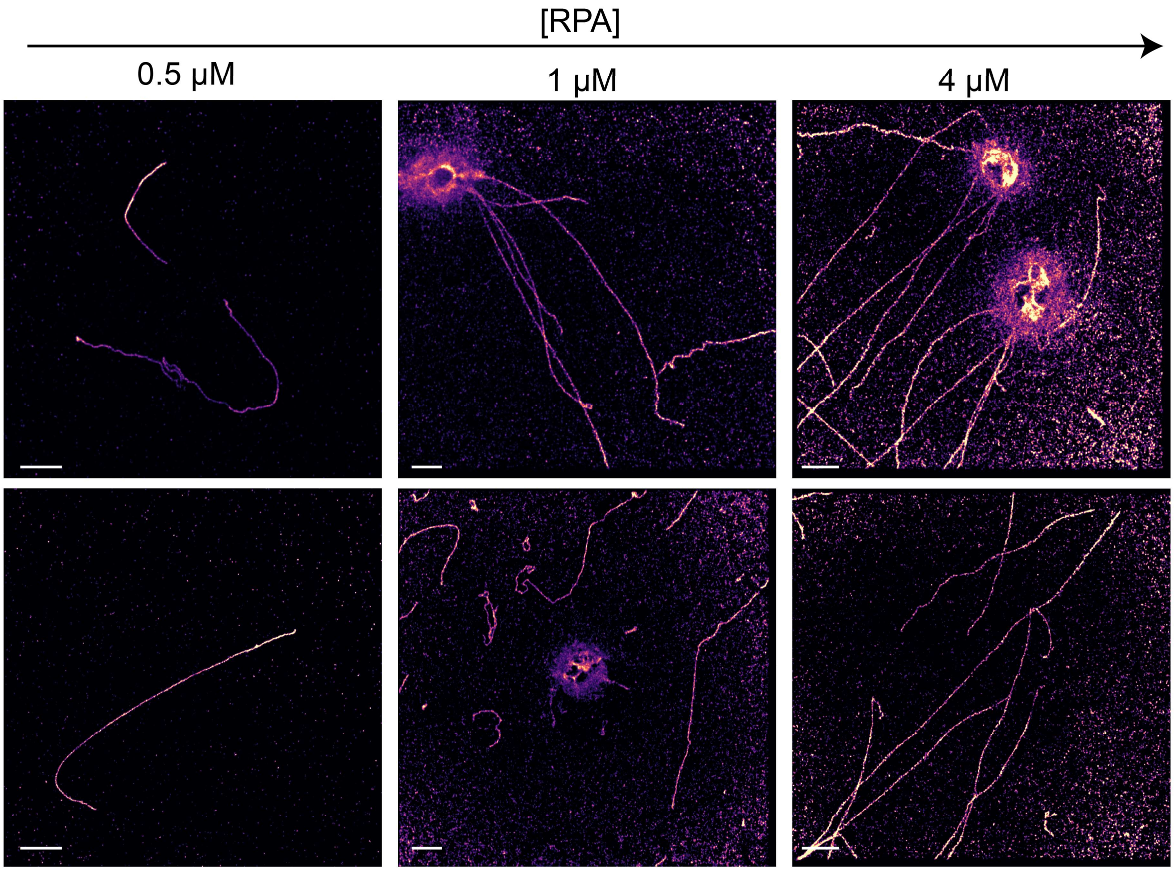


**Figure S11.** Super resolution DNA-PAINT images of RAD51 NPFs formed in the presence of 0.5 µM RAD52 and varying RPA concentration. All scale bars represent are 2 µm.

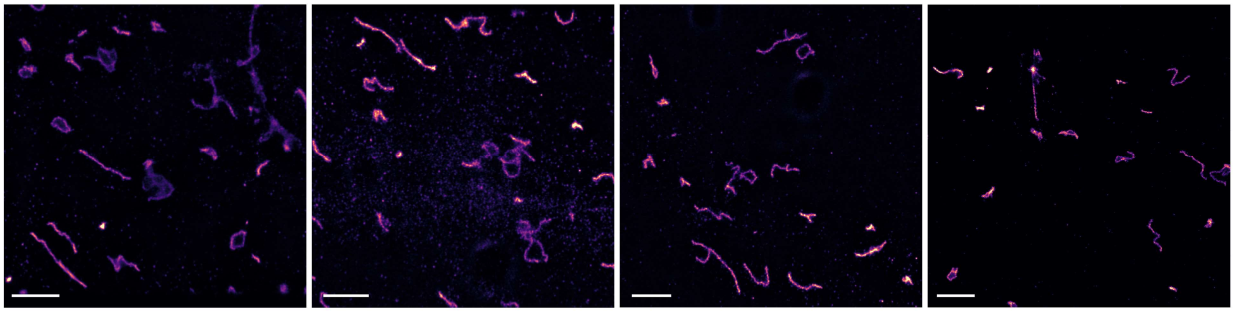

**Figure S12.** Super resolution DNA-PAINT images of RAD51 NPFs formed in the presence of 0.5 μM RAD52 on a $\phi$X174 dsDNA substrate. All scale bars represent are 2 μm.

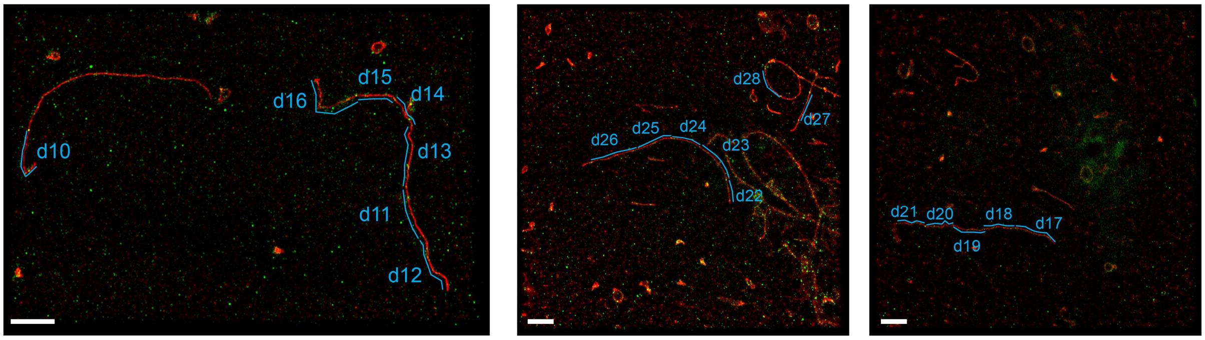


**Figure S13.** Two-color DNA-PAINT images of RAD51 NPFs formed in the presence of 0.5 μM RAD52 on a $\phi$X174 ssDNA substrate with staining for RAD52 (green) and RAD51 (red). All scale bars represent are 2 μm. The segmentation of the long filaments into shorter ones connected by RAD52 spots is used in Figure 6.

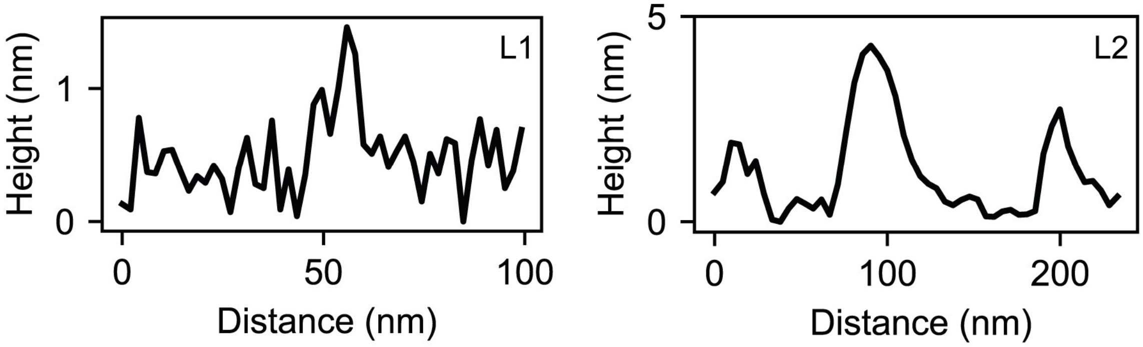


**Figure S14.** Plots showing the measured AFM height of the lines L1 (across a naked ssDNA) and L2 (across a RAD51 NPF) as shown in Figure 7 of the main text. The height of RAD51 NPF (~4 nm) is consistent with the previously reported values in the literature[1].

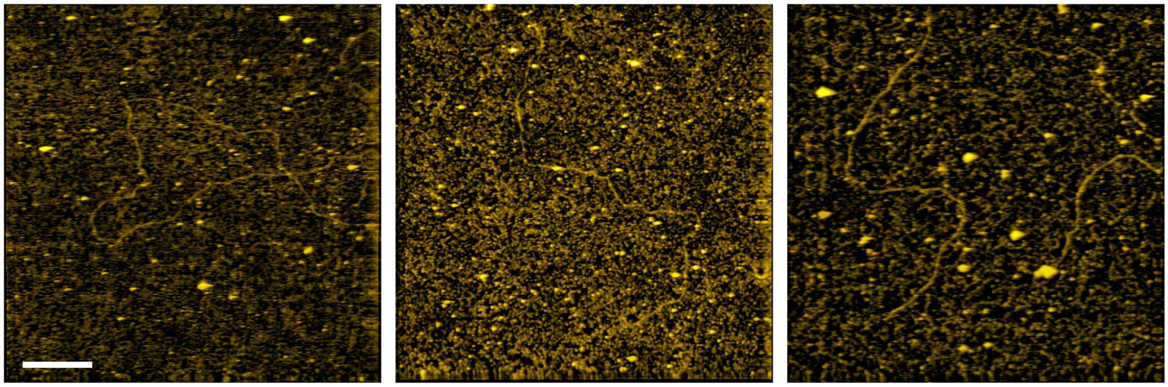

**Figure S15**. AFM images showing long RAD51 filaments that form in presence of RAD52 and RPA. Scale bar is 400 nm.

## References

1 Ristic, D., Kanaar, R. & Wyman, C. Visualizing RAD51-mediated joint molecules: implications for recombination mechanism and the effect of sequence heterology. *Nucleic Acids Research* **39**, 155-167 (2011).
